# Correction: Cerebellar Volumetry in Ataxias: Relation to Ataxia Severity and Duration

**DOI:** 10.1007/s12311-024-01681-2

**Published:** 2024-03-06

**Authors:** Mónica Ferreira, Tamara Schaprian, David Kügler, Martin Reuter, Katharina Deike-Hoffmann, Dagmar Timmann, Thomas M. Ernst, Paola Giunti, Hector Garcia-Moreno, Bart van de Warrenburg, Judith van Gaalen, Jeroen de Vries, Heike Jacobi, Katharina Marie Steiner, Gülin Öz, James M. Joers, Chiadi Onyike, Michal Povazan, Kathrin Reetz, Sandro Romanzetti, Thomas Klockgether, Jennifer Faber

**Affiliations:** 1 German Center for Neurodegenerative Diseases (DZNE), Bonn, Germany; 2 A.A. Martinos Center for Biomedical Imaging, Massachusetts General Hospital, Boston, MA USA; 3 Department of Radiology, Harvard Medical School, Boston, MA USA; 4 Department of Neuroradiology, University Hospital Bonn, Bonn, Germany; 5 Department of Neurology and Center for Translational Neuro- and Behavioral Sciences, University Hospital Essen, University of Duisburg-Essen, Duisburg, Germany; 6 Ataxia Centre, Department of Clinical and Movement Neurosciences, UCL Queen Square Institute of Neurology, London, UK; 7 National Hospital for Neurology and Neurosurgery, University College London Hospitals NHS Foundation Trust, London, UK; 8 Department of Neurology, Donders Institute for Brain, Cognition, and Behaviour, Radboud University Medical Center, Nijmegen, The Netherlands; 9 Neurology Department, Rijnstate Hospital, Arnhem, The Netherlands; 10 Department of Neurology, University Medical Center Groningen, University of Groningen, Groningen, The Netherlands; 11 Department of Neurology, University Hospital Heidelberg, Heidelberg, Germany; 12 Center for Magnetic Resonance Research, Department of Radiology, University of Minnesota, Minneapolis, MN USA; 13 Johns Hopkins University School of Medicine, Baltimore, MD USA; 14 Department of Neurology, RWTH Aachen University, Aachen, Germany; 15 JARA-Brain Institute Molecular Neuroscience and Neuroimaging, Forschungszentrum Jülich, Jülich, Germany; 16 Department of Neurology, University Hospital Bonn, Bonn, Germany; 17 University of Bonn, Bonn, Germany

**Correction: The Cerebellum**

10.1007/s12311-024-01659-0

The original version of this article unfortunately missed to include "Rhenish Friedrich Wilhelm University of Bonn, Germany" as one of the affiliations for the author Monica Ferreira.

With this, the authors has requested that this affiliation will be added to the proof.

The original article has been corrected.

