# Supplementary material for: Cerebellar Volumetry in Ataxias: Relation to Ataxia Severity and Duration
Source: Cerebellum. 2024 Feb 16;23(4):1521–9. doi: 10.1007/s12311-024-01659-0 (PMC11269395; doi:10.1007/s12311-024-01659-0)
Supplement: Supplementary file 1 — (pdf 1065 KB) [file 12311_2024_1659_MOESM1_ESM.pdf]

10. Supplement

10.1. Supplementary descriptive analysis of demographic data

We performed descriptive analysis to identify outliers in the dataset. We used the Tukey's fences method where data points falling below 1.5 times the interquartile range (IQR) below the first quartile or above 1.5 times the IQR above the third quartile are considered as outliers and given special attention. With regard to age, ataxia severity and ataxia duration in total 4 outliers were observed. Three of them were considered as realistic, despite their eccentric location. For one MSA-C patient with an ataxia duration exceeding 20 years the correctness of the clinical diagnosis was questioned. The subject showed a very less affected cerebellum, despite its long disease duration. Thus, this participant was considered as most probably misdiagnosed and therefore excluded from the analyses.

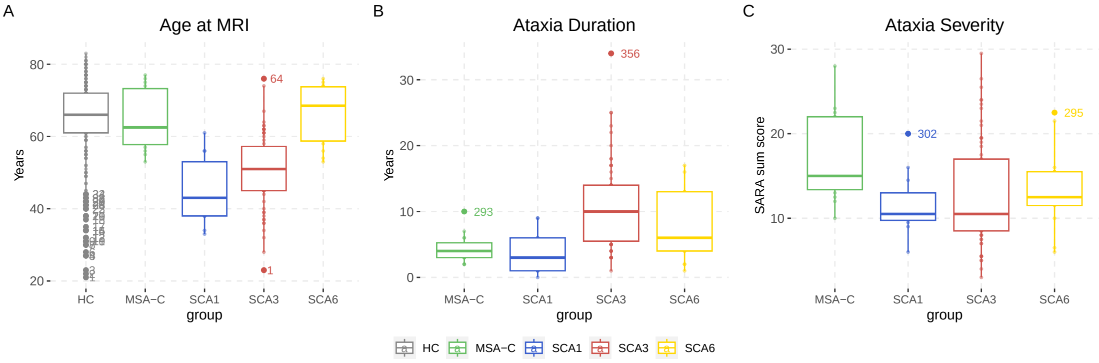

Supplement Figure 1: **Boxplots of age at MRI, ataxia duration and ataxia severity for each ataxia group.** Outliers are indicated as individual data points beyond the whiskers. Age at MRI and and ataxia duration are given in years, ataxia severity is assessed with the Scale for the assessment and rating of Ataxia [13] using the SARA sum score.

10.2. Supplementary descriptive analyses of volume changes in ataxias

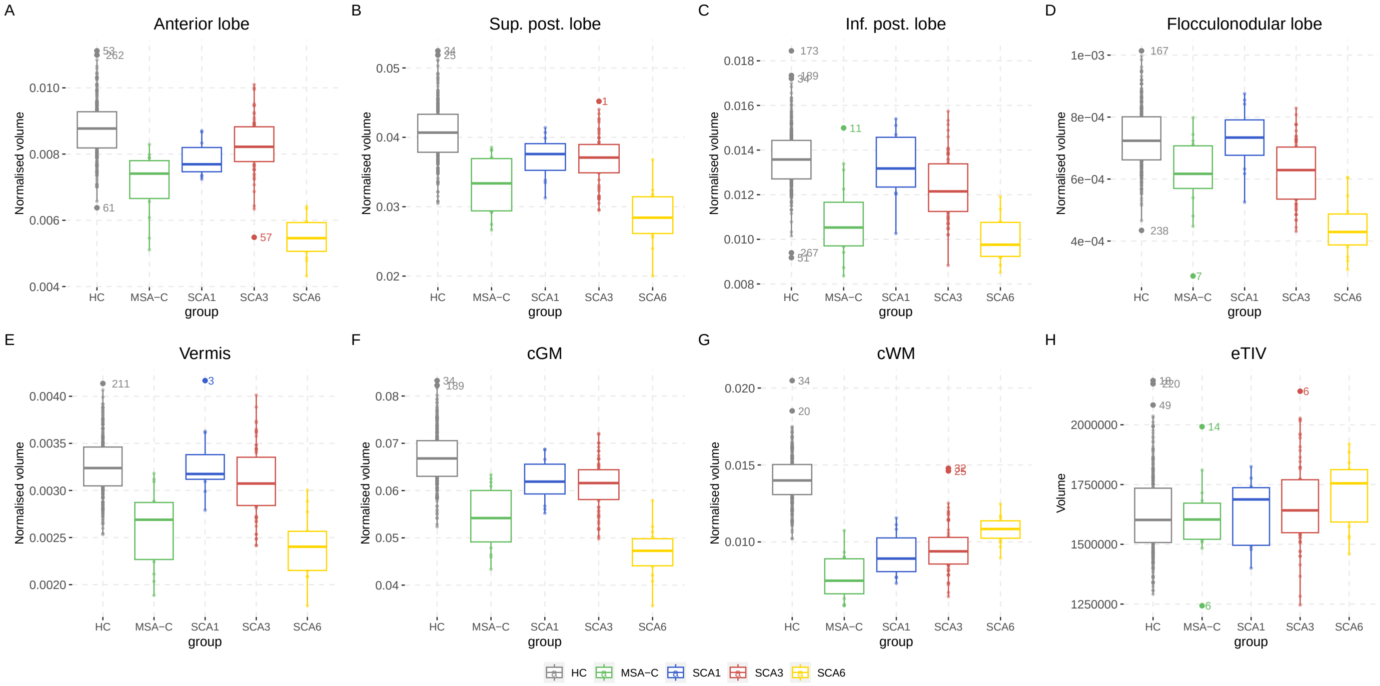

Supplement Figure 2: **Boxplots of each normalised cerebellar volume and the estimated total intracranial volume (eTIV) for each ataxia group.** Outliers are indicated as individual data points beyond the whiskers. cWM - cerebellar whiter matter, cGM - cerebellar grey matter.

Table 1: **Comparison of ataxia patients and age- and sex matched healthy controls.**

Normalised volume	HC vs SCA1	HC vs SCA3	HC vs SCA6	HC vs MSA
Anterior lobe	<.001	<.001	<.001	<.001
Superior Posterior lobe	<.001	<.001	<.001	0.001
Inferior Posterior lobe	0.083	<.001	<.001	0.001
Flocculonodular lobe	0.030	<.001	<.001	0.079
Vermis	0.219	0.003	<.001	0.001
cGM	<.001	<.001	<.001	<.001
cWM	<.001	<.001	<.001	<.001

Comparison of normalised volumes between each ataxia group and HCs was performed after one by one age- and sex-matching of HCs in order to avoid confounding effects. Differences between the resulting matched groups were assessed using a paired t-test. (Table 1).

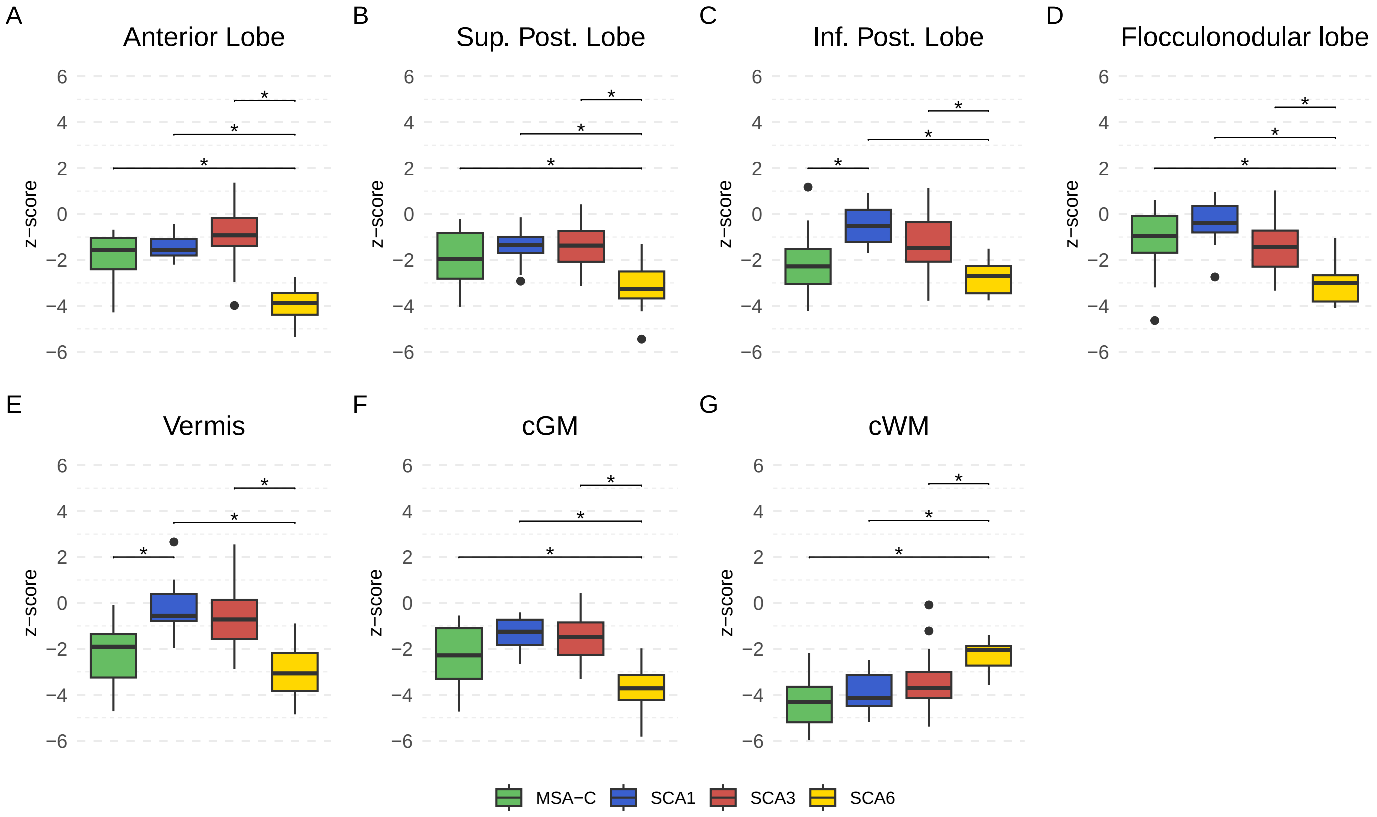

Supplement Figure 3: **Boxplots of the z-scores for SCA1, SCA3, SCA6 and MSA-C in the cerebellar grey and white matter volumes.** Volumes of ataxia patients were z-transformed related to the distribution in the HCs, to account for age-related cerebellar atrophy. Significant differences of the post-hoc analyses with Tukey correction are given for $*p \leq 0.001$. cWM: cerebellar white matter, cGM: cerebellar grey matter.

Linear model evaluation:

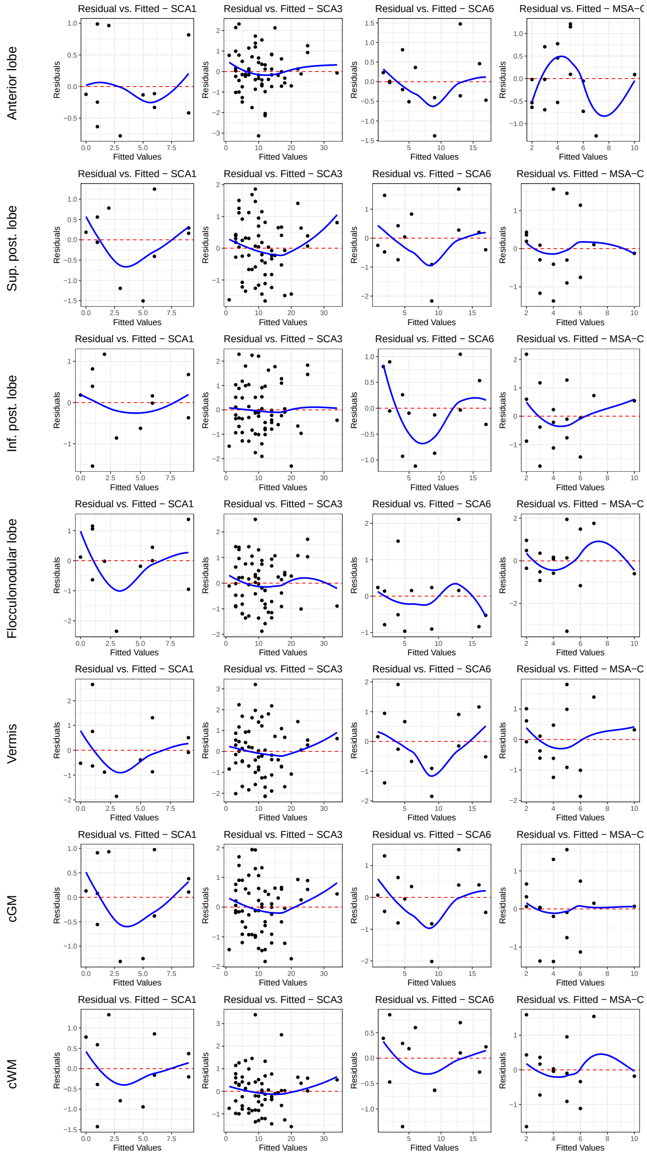

Supplement Figure 4: **Residual versus fitted values for linear relations of z-scores and ataxia duration for each disease.**

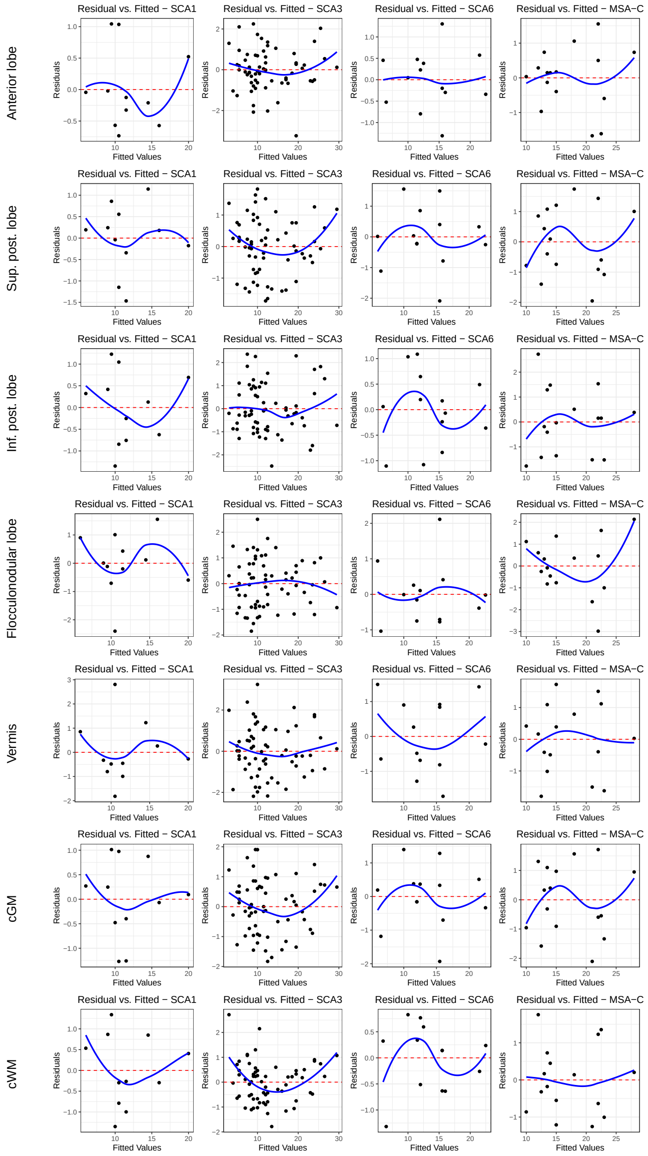

Supplement Figure 5: **Residual versus fitted values for linear relations of z-scores and ataxia severity for each disease.**

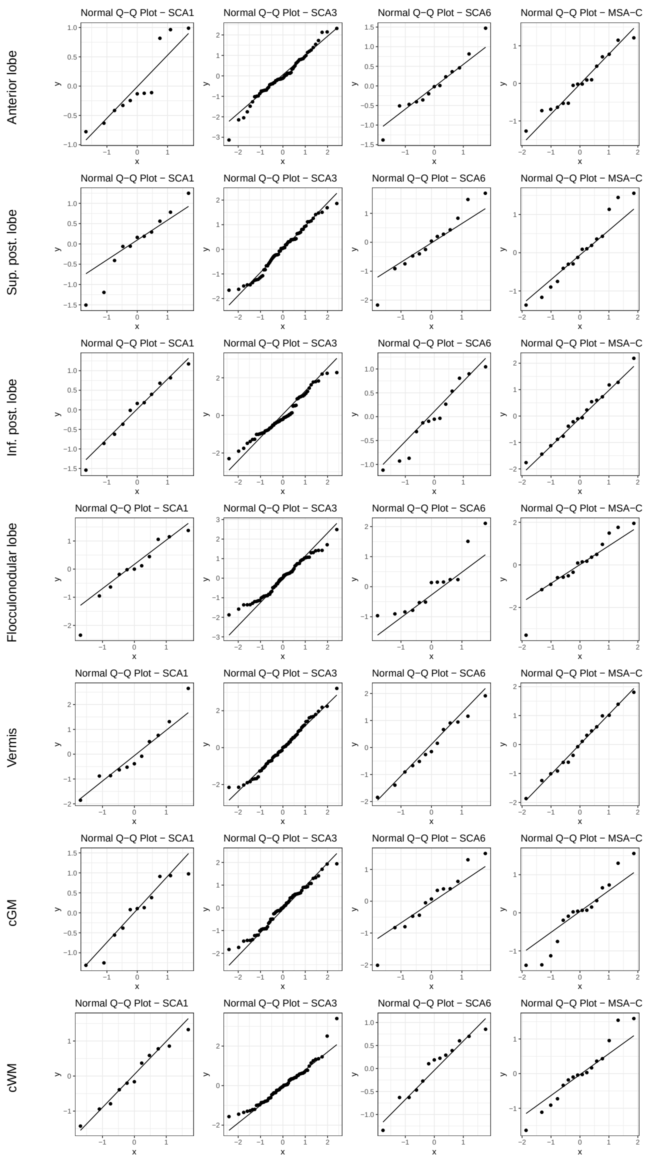

Supplement Figure 6: **Quantile-Quantile (QQ) plots for the residuals of linear relations between z-scores and ataxia duration for each disease**

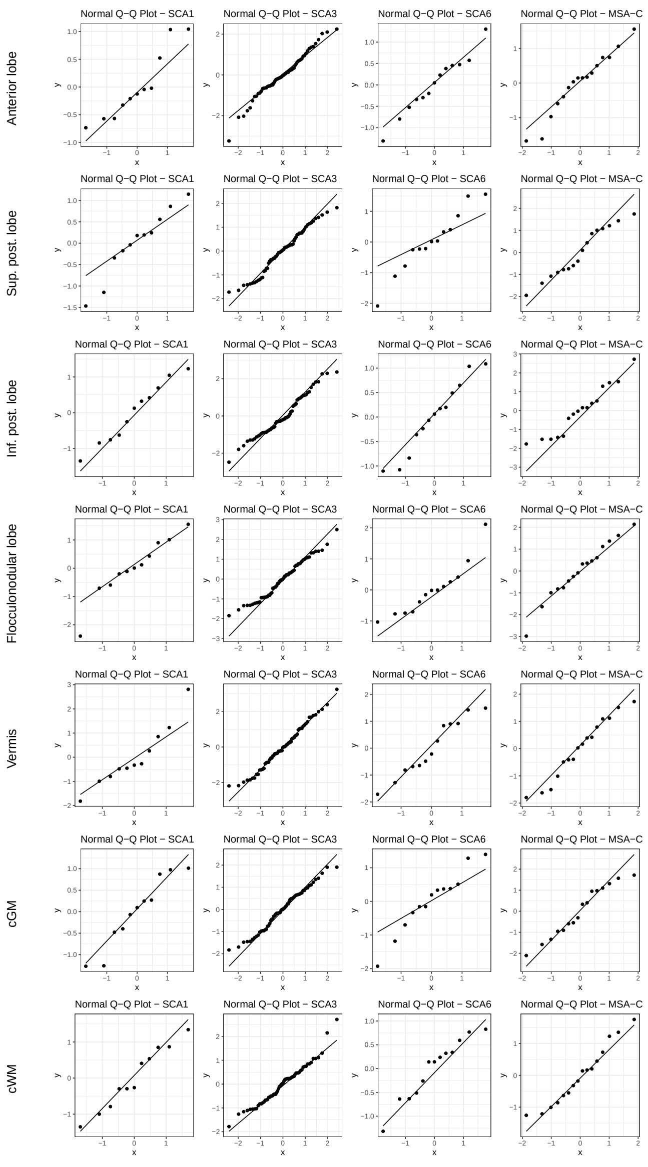

Supplement Figure 7: **Quantile-Quantile (QQ) plots for the residuals of linear relations between z-scores and ataxia severity for each disease**

Quadratic model evaluation:

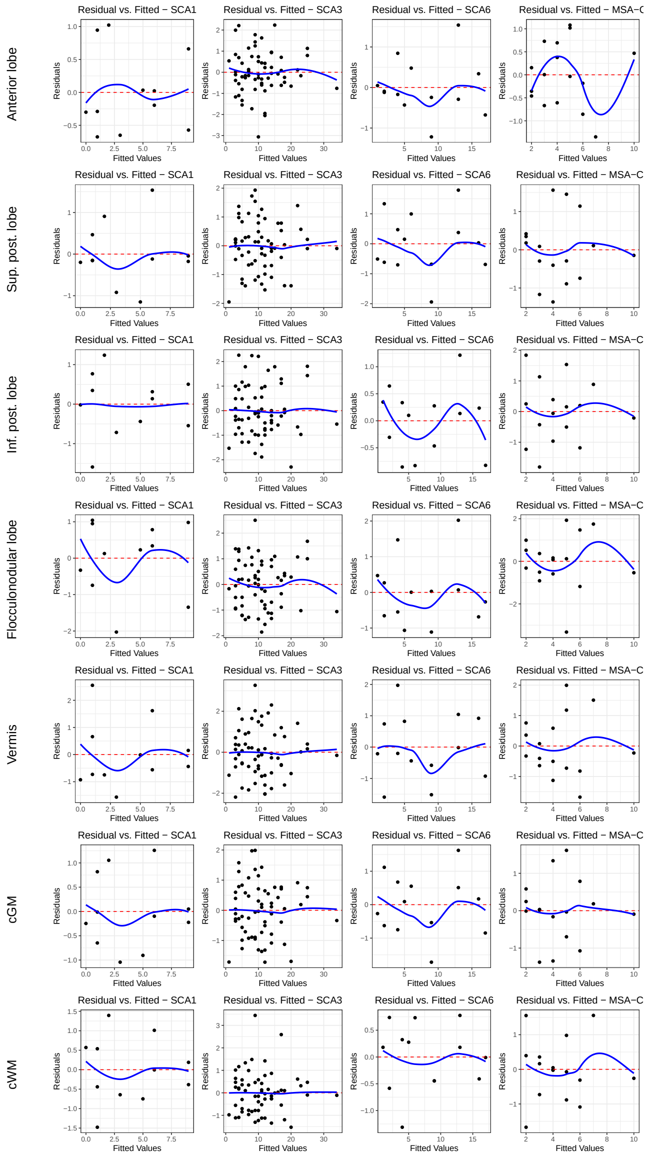

Supplement Figure 8: **Residual versus fitted values for quadratic relations of z-scores and ataxia duration for each disease.**

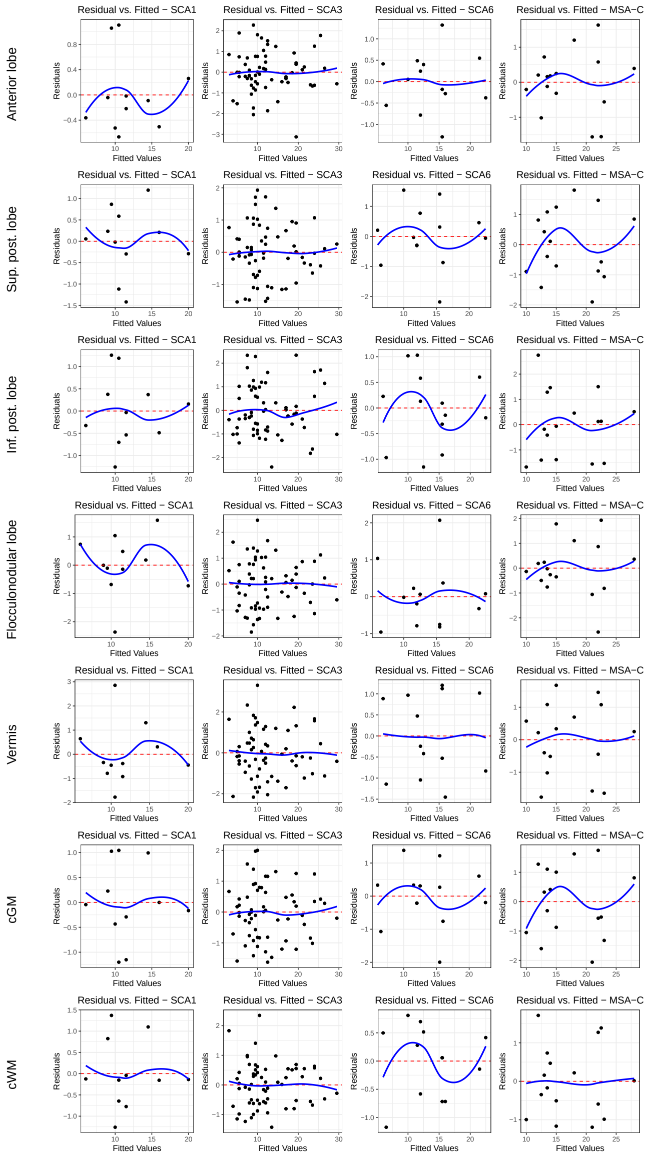

Supplement Figure 9: **Residual versus fitted values for quadratic relations of z-scores and ataxia severity for each disease.**

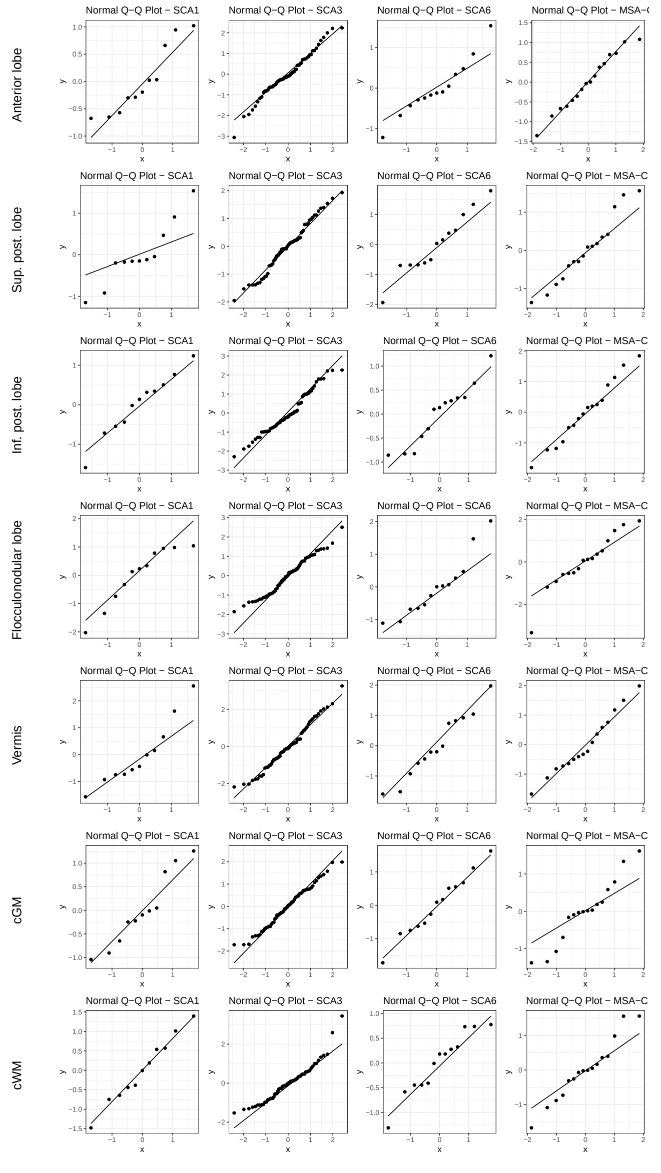

Supplement Figure 10: **Quantile-Quantile (QQ) plots for the residuals of quadratic relations between z-scores and ataxia duration for each disease**

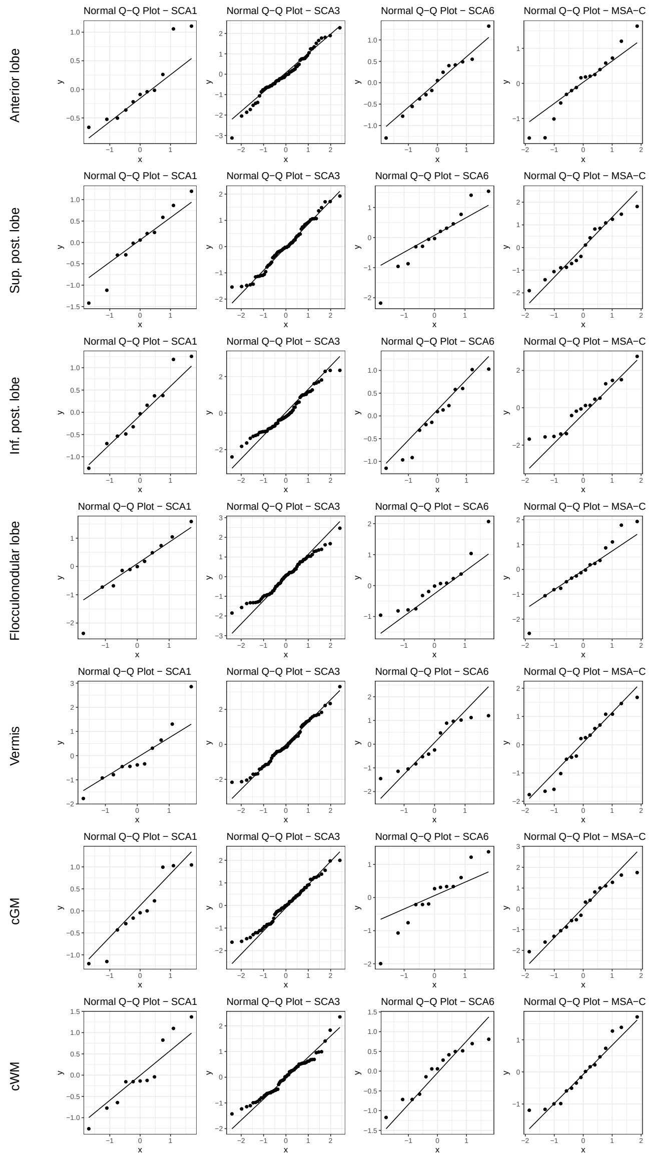

Supplement Figure 11: **Quantile-Quantile (QQ) plots for the residuals of quadratic relations between z-scores and ataxia severity for each disease**

Cubic model evaluation:

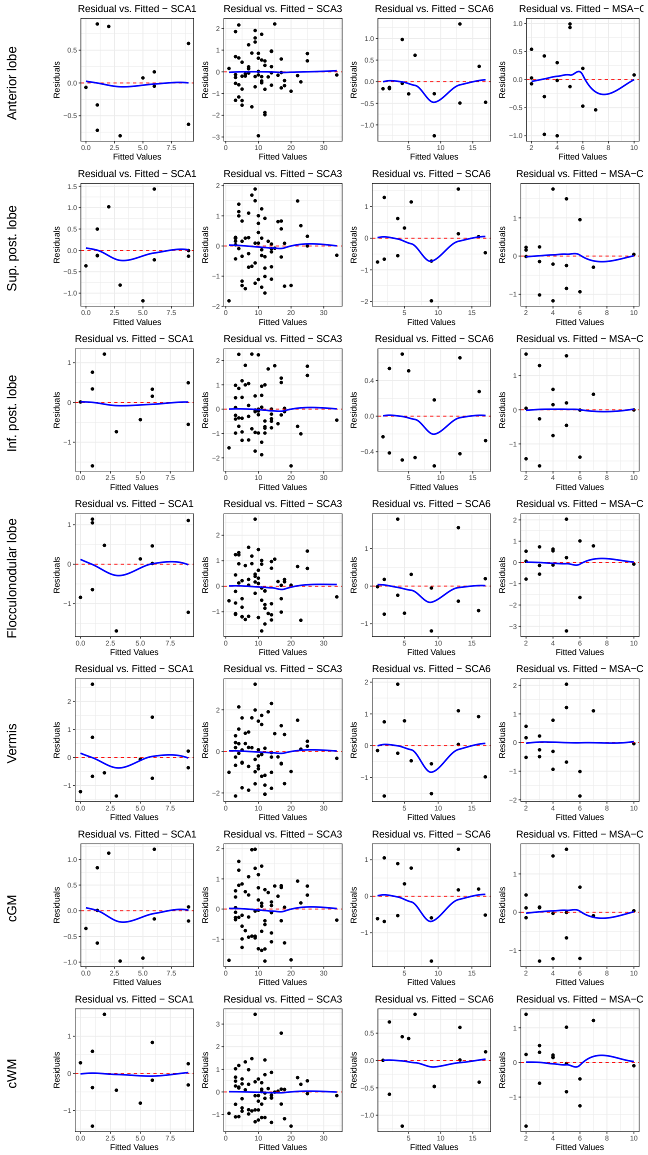

Supplement Figure 12: **Residual versus fitted values for cubic relations of z-scores and ataxia duration for each disease.**

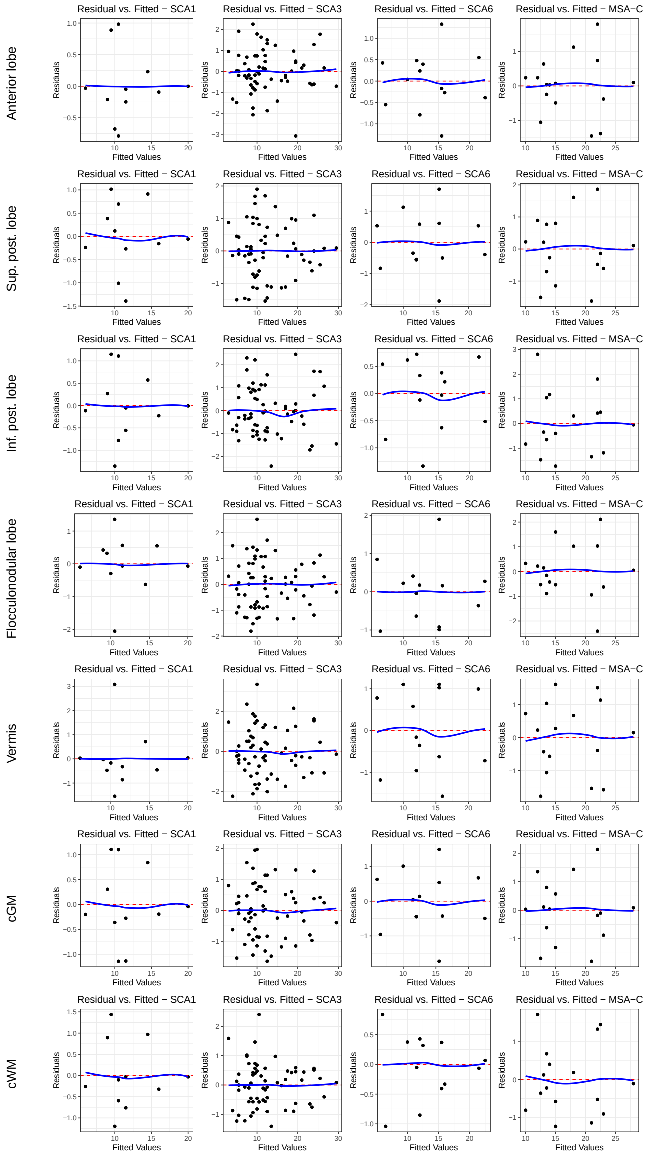

Supplement Figure 13: **Residual versus fitted values for cubic relations of z-scores and ataxia severity for each disease.**

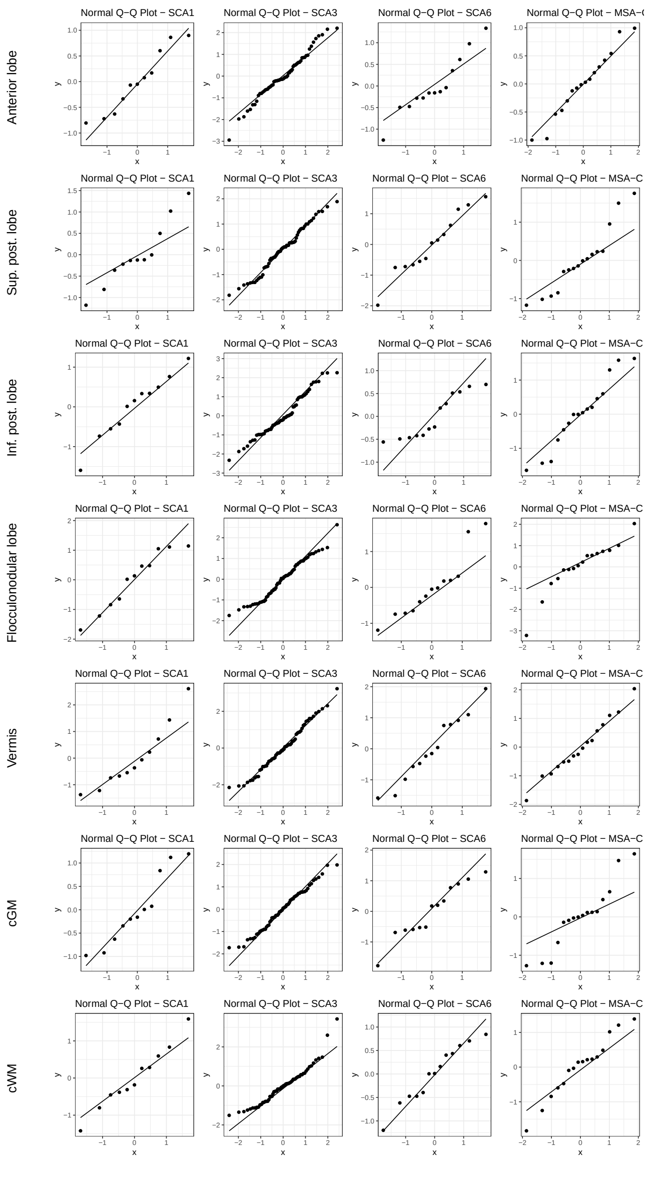

Supplement Figure 14: **Quantile-Quantile (QQ) plots for the residuals of cubic relations between z-scores and ataxia duration for each disease**

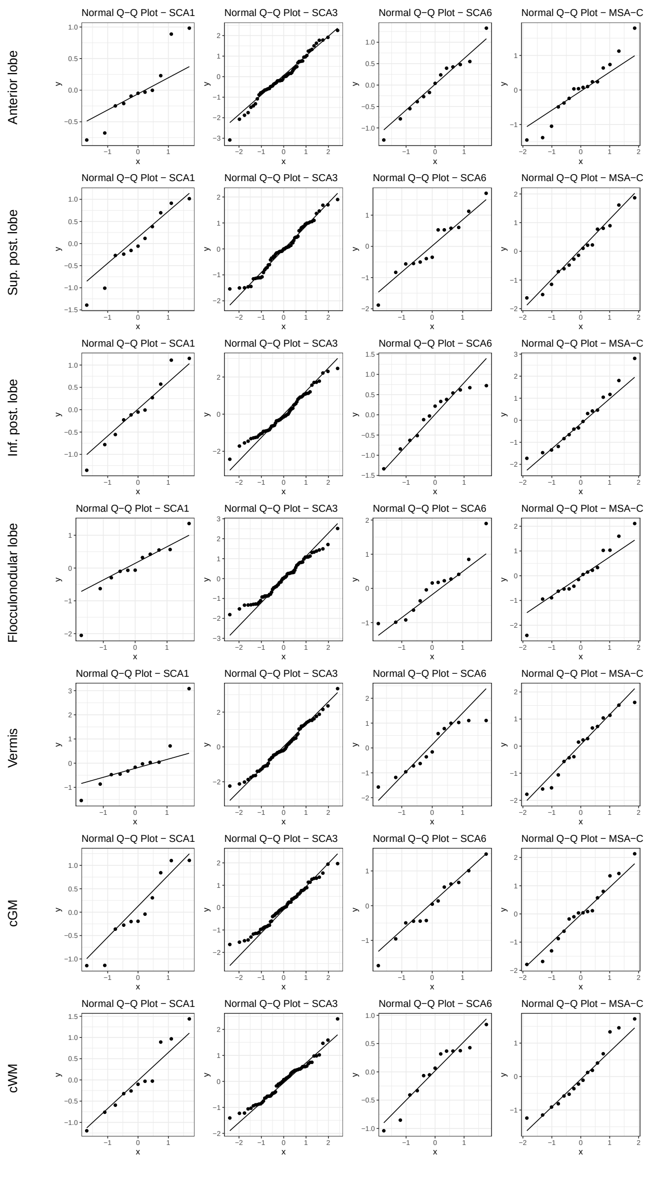

Supplement Figure 15: **Quantile-Quantile (QQ) plots for the residuals of cubic relations between z-scores and ataxia severity for each disease**

Table 2: **Parameters of linear regression model of z-scores with ataxia duration and ataxia severity for each disease.**

		Z-score against ataxia duration				Z-score against ataxia severity			
		Estimate	Std error	t-value	p-value	Estimate	Std error	t-value	p-value
Anterior Lobe	MSA-C	-0.002	0.064	-0.028	0.979	0.034	0.054	0.639	0.538
	SCA1	0.018	0.021	0.067	0.389	0.013	0.021	0.631	0.530
	SCA3	-0.060	0.039	-1.551	0.149	-0.077	0.042	-1.853	0.091
	SCA6	-0.407	0.090	-4.519	$<.001$	-0.132	0.046	-2.881	0.012
Sup. Post. Lobe	MSA-C	0.025	0.082	0.303	0.769	0.056	0.068	0.823	0.432
	SCA1	0.010	0.018	0.537	0.593	-0.009	0.018	-0.487	0.628
	SCA3	-0.070	0.056	-1.241	0.241	-0.089	0.061	-1.448	0.176
	SCA6	-0.356	0.108	-3.285	0.005	-0.039	0.058	-0.677	0.509
Inf. Post. Lobe	MSA-C	-0.107	0.081	-1.313	0.222	-0.067	0.073	-0.924	0.379
	SCA1	-0.021	0.021	-0.969	0.336	-0.011	0.022	-0.495	0.622
	SCA3	-0.059	0.038	-1.529	0.155	-0.056	0.044	-1.259	0.234
	SCA6	-0.442	0.134	-3.306	0.005	-0.105	0.067	-1.559	0.141
Flocculonodular Lobe	MSA-C	-0.002	0.109	-0.021	0.984	-0.045	0.092	-0.486	0.639
	SCA1	0.003	0.019	0.158	0.875	0.006	0.019	0.328	0.744
	SCA3	-0.032	0.051	-0.615	0.551	-0.090	0.052	-1.751	0.108
	SCA6	-0.252	0.161	-1.565	0.140	-0.096	0.067	-1.438	0.172
Vermis	MSA-C	-0.060	0.126	-0.474	0.647	-0.014	0.110	-0.129	0.900
	SCA1	-0.023	0.024	-0.949	0.346	-0.002	0.025	-0.080	0.936
	SCA3	-0.040	0.060	-0.672	0.515	-0.075	0.064	-1.174	0.265
	SCA6	-0.437	0.128	-3.403	0.004	-0.159	0.057	-2.793	0.014
cGM	MSA-C	-0.011	0.083	-0.131	0.899	0.028	0.070	0.397	0.701
	SCA1	0.004	0.018	0.226	0.822	-0.007	0.019	-0.359	0.721
	SCA3	-0.075	0.052	-1.456	0.173	-0.093	0.056	-1.663	0.124
	SCA6	-0.449	0.107	-4.203	0.001	-0.085	0.062	-1.380	0.189
cWM	MSA-C	-0.046	0.087	-0.521	0.615	-0.032	0.075	-0.432	0.676
	SCA1	-0.031	0.018	-1.658	0.102	-0.076	0.017	-4.499	0.000
	SCA3	0.010	0.035	0.296	0.773	0.009	0.039	0.237	0.817
	SCA6	-0.252	0.110	-2.286	0.038	-0.086	0.048	-1.816	0.091
